# Evaluation of local trace element status and 8-Iso-prostaglandin F_2α_ concentrations in patients with Tinea pedis

**DOI:** 10.1186/s12575-015-0030-x

**Published:** 2016-01-05

**Authors:** Meral Miraloglu, Ergul Belge Kurutas, Perihan Ozturk, Ozer Arican

**Affiliations:** Department of Microbiology, Faculty of Medicine, Cukurova University, 01330 Adana, Turkey; Department of Biochemistry, Sutcu Imam University, Faculty of Medicine, Kahramanmaras, Turkey; Department of Dermatology, Sutcu Imam University, Faculty of Medicine, Kahramanmaras, Turkey; Department of Dermatology, Fatih University, Faculty of Medicine, Istanbul, Turkey

**Keywords:** 8-iso-PGF_2α_, Oxidative stress, Trace elements, Tinea pedis

## Abstract

**Background:**

Tinea pedis (TP) is an infection of the feet caused by fungi. The infectious diseases caused by dermatophytes are mainly related to the enzymes produced by these fungi. Up to the now, the local 8-iso-prostaglandin F_2α_ (8-iso-PGF_2α_), concentration as oxidative stress biomarker and trace elements status have not been published in patients with TP. The aim of this study is to evaluate the relationship between oxidative stress and trace elements (Cu, Zn, Se), and to evaluate the ratios of Cu/Zn and Cu/Se in this disorder.

**Methods:**

Forty-three consecutive patients with a diagnosis of unilateral interdigital TP were enrolled in this study. The samples were obtained by scraping the skin surface. 8-iso-PGF_2α_ concentrations in scraping samples were determined by ELISA. In addition, the levels of Se, Zn and Cu in scraping samples were determined on flame and furnace atomic absorption spectrophotometer using Zeeman background correction.

**Results:**

Oxidative stress was confirmed by the significant elevation in 8-iso-PGF_2α_ concentrations (*p* < 0.05). When compared to non-lesional area, Zn and Se levels were significantly lower on lesional area, whereas Cu levels was higher on the lesional area than the non-lesional area (*p* < 0.05). In addition, the correlation results of this study were firstly shown that there were significant and positive correlations between Cu and 8-iso-PGF_2α_ parameters, but negative correlations between Se-Cu; Se-8-iso-PGF_2α_ parameters in lesional area. Furthermore, the ratios of Cu/Zn and Cu/Se were significantly higher on the lesional area than the non-lesional area (*p* < 0.05). According to sex and fungal subtypes, there was no significant difference in the concentrations of 8-iso-PGF_2α_ and trace elements in patients with TP (*p* > 0.05).

**Conclusions:**

Our results showed that there is a possible link between oxidative stress (increased 8-iso-PGF_2α_ concentrations) and imbalanced of trace elements status in lesional area of TP patients. The use of antifungal agents together with both Zn and Se drugs could be helpful in the both regression of disease and in shortening the duration of disease.

## Background

Dermatophyte infections are common disorders worldwide, and the incidence of their has increased considerably during the past several decades [1]. Tinea pedis (TP), which is a dermatophytic infection of the feet, can involve the interdigital web spaces or the sides of the feet and may be a chronic or recurring condition [2]. It is generally confined to the stratum corneum in the epidermis and cutaneous appendages [3]. The ability of certain fungi to adhere to a particular host arises from numerous mechanisms and host factors, including the ability to adapt to the human body [4]. The physical and chemical structure of the skin represents a form of defense against fungal pathogens [5]. Neutrophils and monocytes/macrophages appear to be important in the defense against fungi, including those involved in the cutaneous mycoses. Neutrophils can directly attack pathogens by a variety of microbicidal processes [6]. The neutrophil oxidative mechanisms are capable of killing *Trichophyton sp*. in vitro [7], [8], suggesting that they could have a role in the defense against dermatophytosis. This mechanism may be active against the organisms causing superficial fungal infections also [6].

Reactive oxygen species (ROS) are generated by cells under certain physiological conditions. Insufficient antioxidant protection or excess production of ROS cause oxidative damage. The balance between oxidative damage and antioxidant enzyme systems appears to determine the physiological and pathological effects of ROS. Depending on the damage of skin, the free radicals are increased and so that this causes the lipid peroxidation (LPO). Three LPO products, malondialdehyde (MDA), 4-hydroxynonenal (HNE), and 8-iso-prostaglandin F_2α_ (8-iso-PGF_2α_), play the role the pathophysiology of human diseases [9]. Oxidative modification of arachidonic acid leads to the formation of free radical-catalyzed products called F_2_-isoprostanes [10]. One of these compounds, 8-iso-PGF_2α_, has recently been shown to be a specific, chemically stable, quantitative marker of oxidative stress in vivo. Therefore, to assess the importance of oxidative stress in the progression to TP patients, we measured, using a specific validated immunoassay in scraping samples, concentrations of 8-iso-PGF_2α_ in a series of consecutive patients with TP. It was reported that 8-iso-PGF_2α_’s concentration increased in the various diseases [11]. Trace elements should present in the body in appropriate amounts and must be available for reacting with other elements to form critical molecules as well as to participate in various important chemical reactions. This fact suggests that various immunological and inflammatory changes associated with physiological and pathological conditions can affect trace element distribution in the body. Thus, Cu/Zn could potentially represent one of the most sensitive clinical markers of these changes [12, 13].

Effector mechanisms in dermatophytes, that contribute to the killing of fungi in the epidermis are poorly understood [8]. To our knowledge, there has been no study published on local trace elements status and 8-iso-PGF_2α_ concentrations, and Cu/Zn and Cu/Se ratios in patients with TP. Therefore, we aimed to investigate trace elements and 8-iso-PGF_2α_ concentrations in patients with TP, and to clarify association between oxidative stress and trace elements in skin with pathogenic fungi may be helpful to find possible targets for development of new antifungal drugs.

## Results

Forty-three patients were included in the study. Twenty-nine were female (54.5 %) and 14 were male (44.5 %). The mean age of patients was 40.0 ± 10.2 years (range, 21–58.0). The mean disease duration was 4.2 ± 1.0 months (range, 3–6.0).

Strains of *Trichophyton rubrum* (55), *Trichophyton mentagrophytes* (35) and *Epidermophyton floccosum* (10 %) were found from the cultures.

When compared to non-lesional area, Zn and Se levels were significantly lower in lesional area, whereas Cu levels was higher in lesional area than those in non-lesional area (*p* < 0.05). Furthermore, the ratios of Cu/Zn and Cu/Se were significantly higher in lesional area than the non-lesional area (*p* < 0.05). These results were seen in Table 1. In addition, there were significant positive and negative correlations between trace elements and 8-iso-PGF_2α_ concentrations in lesional and non-lesional areas as shown in Table 2. Our study shows that oxidative stress is increased in lesional area as reflected by elevated 8-iso-PGF_2α_ concentrations (*p* < 0.05). Furthermore, we found that there was no difference among the concentrations of 8-iso-PGF_2α_ and trace elements in patients with TP according to sex and fungal subtypes (*p* > 0.05) (Not shown data).

**Table 1:** 8-iso-PGF_2α_concentrations as oxidative stress biomarker and trace elements in lesional and non-lesional areas

	Lesional area mean ± SD (min–max)	Non-Lesional area mean ± SD (min–max)
8-iso-PGF_2α_(pg/mL)	47.6 ± 9.5 (34.2–59.1)	11.1 ± 1.5 (9.7–23.3)
Zn (mg/L)	**35.08 ± 10.01 (29.0–53.0)	63.17 ± 11.45 (43.0–72.0)
Se (mg/L)	**23.05 ± 4.27 (19.0–42.0)	45.07 ± 8.91 (33.2–51.0)
Cu (mg/L)	**97.07 ± 8.89 (54–103)	67.84 ± 18.70 (55.0–77.0)
Cu/Zn	***2.76 ± 0.93 (2.2–3.0)	1.07 ± 0.52 (0.7–1.3)
Cu/Se	***4.21 ± 2.45 (3.6–6.0)	1.50 ± 0.8 (1.2–2.0)

SD: standard deviation

*Significant differences in the levels of 8-iso-PGF_2α_as oxidative stress biomarker in lesional and non-lesional areas (*p* < 0.05)

**Significant differences in the levels of trace elements such as Zn, Se and Cu in lesional and non-lesional areas (*p* < 0.05)

***Significant differences in the ratios of Cu/Zn and Cu/Se in both areas (*p* < 0.05)

**Table 2:** The correlation between trace elements and 8-iso-PGF_2α_concentrations as oxidative stress biomarker

		Zn	Se	Cu	8-iso-PGF_2α_	Cu/Zn
Se	R	−0.384				
	P	0.180				
Cu	R	−0.038	−0.689			
	P	0.807	0.018*			
8-iso-PGF_2α_	R	0.230	−0.798	0.785		
	P	0.503	0.009*	0.009*		
Cu/Zn	R	−0.101	−0.685	0.857	0.902	
	P	0.784	0.067	0.002*	0.001*	
Cu/Se	R	0.134	−0.850	0.907	0.902	0.901
	P	0.685	0.002*	0.001*	0.001*	0.005*

r: The correlation coefficient method. Pearson correlation was used

*There was significant correlation between 8-iso-PGF_2α_ concentrations and trace elements (*p* < 0.05)

## Discussion

To our knowledge, this is the first study examining the concentrations of 8-iso-PGF_2α_ and trace elements status demonstrated in infected human skin surface with dermatophytosis. These results indicated that there were important evidence of Zn, Se and Cu levels on the pathophysiology of TP.

Neutrophils can directly attack pathogens by a variety of microbicidal processes, and they could have a role in the defense against dermatophytosis. Also, they generate ROS such as superoxide anion, hydrogen peroxide and hydroxyl radicals resulting in damaged proteins, lipids, DNA, and destroying phagocytosed pathogens. Macrophages have an additional antimicrobial mechanism by which they can use production of nitric oxide to inhibit growth of ingested fungal pathogens, such as *Cryptococcus neoformans*. This mechanism may be active against the organisms causing superficial fungal infections also [14]. In pathological conditions, the human body usually has adequate reserves against the production of free radicals, which are produced during metabolism. However, when free radical generation exceeds the antioxidant production capacity, oxidative stress occurs in various diseases [15]. Recently, there has been considerable interest in oxidative stress caused by ROS and its involvement in disease processes [16, 17]. The results of the present study showed that 8-iso-PGF_2α_ (lipid peroxidation product) level was significantly higher in lesional area compared to non-lesional area. Our results suggest that elevated 8-iso-PGF_2α_ concentration in an infected area with fungus may lead to oxidative stress due to impaired of antioxidant defence system. Some authors reported [18, 19] increased lipid peroxidation levels in dermatophytic animal studies. However, there is one study in humans. Ozturk et al. reported that lipid peroxidation levels in scraping sample of TP patients increased more than control group as similar to our study [20]. This may be an indication of oxidative stress derived from inflammation in this lesional area.

Trace elements are required in small concentrations as components of these antioxidant enzymes. Cytoplasmic superoxide dismutase (SOD) enzyme contains Cu and Zn metals as cofactors; glutathione peroxidase (GSH-Px) enzyme contains Se and catalase contains Fe [21]. It is demonstrated that these trace element concentrations are subject to change in several disease problems [22]. Cu is an imperative molecule in life; in contradiction, however, it is highly toxic [22], [23]. Cells have highly specialized and complex systems for maintaining intracellular Cu concentrations. In our study, Cu levels were found to be positively correlated with 8-iso-PGF_2α_.concentrations Cu causes lipid peroxidation as enhancing the formation of hydroxyl radical [24]. We thought that Cu may cause oxidative stress coupled with the 8-iso-PGF_2α_. Also, increased Cu concentrations on lesional area may also arise from the release of Cu during inflammatory tissue damage. Zn is a mineral that plays a vital role in many biological processes, such as enzyme action, cell membrane stabilization, gene expression and cell signalling [25, 26] . It is required for structural and functional integrity of more than 2000 transcription factors and 300 enzymes; hence, almost all metabolic pathways are in some ways reliant on at least one Zn requiring protein [27, 28]. In addition, Zn is also an integral part of key antioxidant enzymes and Zn deficiency impairs their synthesis, resulting in increased oxidative stress [29]. Clinically, both systemic and local applications of zinc have given the successful results [30]. Zn could be a physiological part of the antioxidant defence system [31]. The antioxidant function of it may be related to several factors. First, Zn is an essential component of SOD. The second potential mechanism for its antioxidant effects is the antagonism of redox-active transition metals, and the prevention of oxidation of sulfhydryl groups within proteins. As the third potential antioxidant mechanism of Zn is considered the regulation of metallothione in metabolism [32]. In the present study, the decreased in Zn level in lesional area of TP subjects may be related to increased intestinal absorption of Cu due to Zn deficiency. Se functions in the antioxidant system are an essential component of a family of GPx enzymes. Deficiency of GPx may occur in the presence of severe Se deficiency [33]. In our study, Se levels in lesional area was significantly lower than non-lesional area. The reduction in Se levels might be due to the increased consumption of these agents by the erythrocytes and/or other tissues in response to increased oxidative stress in TP patients. Some authors reported that decreased Cu, Zn and Se levels in dermatophytic animal studies [19]. Because there is no human study, we could not make any comparison. Furthermore, we found that there was no difference among the concentrations of 8-iso-PGF_2α_ and trace elements in patients with TP according to sex and fungal subtypes. Normally, some trace element parameters and 8-iso-PGF_2α_ levels in human plasma or serum are not affected by sex (e.g. selenium) [34].

We did not use Zn or Se application as a therapeutic option in our patients. We speculated that new studies of the topical applications of Zn and Se may explain the imbalanced of trace elements status in TP. These results may be helpful in the regression of disease or in shortening the duration of disease.

We found that the Cu/Zn and Cu/Se ratios were higher in lesional area compared to non-lesional area, and Cu/Zn ratios were positively correlated with Cu concentrations. Changes in levels of Cu and Zn have also been demonstrated for certain diseases, but an imbalance of the Cu/Zn ratio seems to be a better indicator of infection, vascular complications, and prognosis of diseases than Zn or Cu status alone [24]. In our study, increased levels of Cu/Zn ratios may due to high Cu levels. We thought that elevated Cu levels, Cu/Se and Cu/Zn ratios may initiated a lipid peroxidation and this situation can result in lesion.

## Conclusion

The present results showed that there is a possible link between oxidative stress (increased 8-iso-PGF_2α_ concentrations) and imbalanced of trace elements status in lesional area of TP patients. Use of antifungal agents together with both Zn and Se drugs seems to reasonable in treatment of TP patients.

## Methods

The study was approved by the local ethical committee of Sutcu Imam University, Medical Faculty, Kahramanmaras, Turkey. Prior to the initiation of the study, each subject was informed about the aim of the study and signed an informed consent form. The data collection of the study was performed over 6 months, January to July 2015. TP was diagnosed on the basis of history, physical examination, native preparation and fungal culture. All TP patients who had local treatment resistant and frequently relapsing, and also they had not used any topical or systemic treatment for the last 3 months. Only interdigital macerated TP was included in our study. Vesicle, bulla, pustular lesions were excluded. Patients with systemic disease such as cardiac, renal and endocrine were excluded. Onychomycosis, other subtypes of TP and combined type TP were not included in our study either. Erythrasma and tinea versicolor were excluded by Wood’s lamp. Bacterial infection was excluded with bacterial cultures. Thirty-six consecutive patients with a diagnosis of unilateral TP were enrolled into the study. At the same time, because of invisible perilesional dermatomycotic infection, the uninfected symmetrical interdigital area of the other foot was preferred as the non-lesional skin of patients (KOH examination, fungal cultures and Wood’s lamp were negative). Samples were obtained from a moderate area in the skin with a sterile scalpel by scraping the edges of the lesions and placed in sterile Petri dishes. Then, the samples were examined by direct microscopy, samples were cultured in Sabouraud’s dextrose agar (SDA) medium. For direct microscopic examination of samples, skin scraping samples were brought to the laboratory, one or two drops of 15 % KOH solution was dropped onto a clean slide, and samples were placed on the slide and closed with a lamella. Samples were stored at room temperature for 15–20 min, then x40 magnification microscopic examination was performed and arthrospores, budding and blastospores were investigated. The examples in which fungal elements were observed were considered as positive, others considered as negative.

### Planting and culture of the samples

Plantings were performed by the method of drilling three into SDA medium. Cultures were checked 2–3 times a week during incubation. Two cultures were kept at room temperature and one culture was kept at 37 °C for 4 weeks when they were evaluated. Cultures in which reproduction was not determined were considered negative. Identification of fungal colonies grown in culture was achieved by examining the macroscopic and microscopic structure.

### Macroscopic examination of the colony

At the end of the incubation period, cultivated cultures were evaluated by the surface (aerial mycelial) and the base color, surface lattice (bare, waxy, powdery, granular, suede-like, velvety, furry, fluffy), topography (flat, raised, dispersed), the colony’s meandering shape (the radially, the brain, such as craters) and evaluated by the speed of reproduction.

### Microscopic examination of the colony

This investigation was performed to determine the presence of macroconidia and microconidia, and to examine the structures of hyphae. Furthermore, Wood’s examination was done to look for erythrasma on both feet of all patients to rule out any possibility of *Corynebacterium minutissimum* infections. Bacteriological culture was performed to rule out bacterial infections. The patients had no history of any topical and systemic drug therapy including vitamins, iron and anti-inflammatory drugs for at least 3 months, and none of them had any other coexistent systemic and cutaneous diseases. None of them had alcohol abuse problems or smoked. No female patients were pregnant. Regular sports and heavy trades workers were not included in the study.

### Biochemical analysis

#### Preparation of scraping homogenates

Infected keratinous tissue was taken non-dissolved from fungal elements. These scraping samples were homogenized in two volumes (w/v) of the 1.15 % ice-cold KCl solution, using a Heidolph 50110 R2R0 homogenizer (Schwabach, Germany). Biochemical assays were performed on the supernatant preparation in a Sorvall RC-2B (Minneapolis, MN, USA) centrifugation of the homogenate at 39 880 g for 30 min at 4 °C.

### Measurement of 8-Iso-PGF_2α_

This assay employs the quantitative sandwich enzyme immunoassay technique. Antibody specific for 8-iso-PGF_2α_ has been pre-coated onto a microplate. Standards and samples are pipetted into the wells and any 8-iso-PGF_2α_ present is bound by the immobilized antibody. After removing any unbound substances, a biotin-conjugated antibody specific for 8-iso-PGF_2α_ is added to the wells. After washing, avidin conjugated Horseradish Peroxidase (HRP) is added to the wells. Following a wash to remove any unbound avidin-enzyme reagent, a substrate solution is added to the wells and color develops in proportion to the amount of 8-iso-PGF_2α_ bound in the initial step. The color development is stopped and the intensity of the color is measured at 450 nm.

### Measurement of trace elements

#### Measurement of Se level

Se measurement in scraping samples was done in graphite furnace atomic absorption spectrophotometer (Perkin Elmer Analyst 800) using Zeemanbackground correction. Matrix modifiers were palladium (4 mg in20-mL sample) and Mg sulfate (3 mg in 20-mL sample). Samples and calibration standards were diluted in 1:3 with 0.05 % Triton X-100 to improve the sample viscosity and reproducibility ofthe results. Se levels in all groups were evaluated according to astandard curve. Se calibration standards were prepared from thecommercial Se standard (1000 mg/L) by serial dilutions [35].

#### Measurement of Cu levels

Cu levels in scraping samples were analyzed in flame photometer of atomic absorption spectrophotometer (Perkin Elmer Analyst 800). Samples and calibration standards for Cu measurement were 1:2 dilutions with 10 % glycerol. Commercial Cu calibrators were used asstandards (1.000 mg/L) by serial dilutions and samples were evaluated according to a standard curve [36].

#### Measurement of Zn levels

Zn levels in scraping samples were analyzed in flame photometer of atomic absorption spectrophotometer (Perkin Elmer Analyst 800). Samples and calibration standards for Zn measurement were prepared in 1:4 dilutions with 5 % glycerol. Commercial Zn standards (1.000 mg/L) were used by serial dilutions and samples were evaluated according to standard curve [37].

### Statistical analysis

Statistical analysis was carried out using SPSS 15.0 for Windows statistical software. The conformability of the quantitative data to the normal distribution was examined by the Kolmogorov–Smirnov test. The paired Student’s t-test was used to compare mean values for all parameters between lesional and non-lesional skin. The Mann–Whitney U-test was used to compare mean values according to sex. Pearson correlation was used for parameters between lesional and non-lesional areas. Also, one-way ANOVA was used to determine differences among subtypes. *p* < 0.05 was considered statistically significant.

## Abbreviations

TP: Tinea pedis
ROS: Reactive oxygen species
LPO: Lipid peroxidation products
MDA: Malondialdehyde
HNE: 4-hydroxynonenal
8-iso-PGF_2α_: 8-iso-prostaglandin F_2α_
SDA: Sabouraud’s dextrose agar
HRP: Horseradish peroxidase
GSH-Px: Glutathione peroxidase
SOD: Superoxide dismutase
